# A qualitative analysis of environmental policy and children's health in Mexico

**DOI:** 10.1186/1476-069X-9-14

**Published:** 2010-03-23

**Authors:** Enrique Cifuentes, Leonardo Trasande, Martha Ramirez, Philip J Landrigan

**Affiliations:** 1 Environmental Health, Center for Population Health Research, National Institute of Public Health (INSP), Universidad No 655, Col Santa María Ahuacatitlán, Cerrada Los Pinos y Caminera Cuernavaca, Morelos CP 62100, México; 2 Department of Preventive Medicine, Mount Sinai School of Medicine, New York, 1 Gustave L Levy Place, Box 1057, New York, NY 10029, USA; 3 Environmental Health, Harvard University School of Public Health, Boston, MA, USA

## Abstract

**Background:**

Since Mexico's joining the North American Free Trade Agreement (NAFTA) and the Organization for Economic Cooperation and Development (OECD) in 1994, it has witnessed rapid industrialization. A byproduct of this industrialization is increasing population exposure to environmental pollutants, of which some have been associated with childhood disease. We therefore identified and assessed the adequacy of existing international and Mexican governance instruments and policy tools to protect children from environmental hazards.

**Methods:**

We first systematically reviewed PubMed, the Mexican legal code and the websites of the United Nations, World Health Organization, NAFTA and OECD as of July 2007 to identify the relevant governance instruments, and analyzed the approach these instruments took to preventing childhood diseases of environmental origin. Secondly, we interviewed a purposive sample of high-level government officials, researchers and non-governmental organization representatives, to identify their opinions and attitudes towards children's environmental health and potential barriers to child-specific protective legislation and implementation.

**Results:**

We identified only one policy tool describing specific measures to reduce developmental neurotoxicity and other children's health effects from lead. Other governance instruments mention children's unique vulnerability to ozone, particulate matter and carbon monoxide, but do not provide further details. Most interviewees were aware of Mexican environmental policy tools addressing children's health needs, but agreed that, with few exceptions, environmental policies do not address the specific health needs of children and pregnant women. Interviewees also cited state centralization of power, communication barriers and political resistance as reasons for the absence of a strong regulatory platform.

**Conclusions:**

The Mexican government has not sufficiently accounted for children's unique vulnerability to environmental contaminants. If regulation and legislation are not updated and implemented to protect children, increases in preventable exposures to toxic chemicals in the environment may ensue.

## Introduction

The past two decades have witnessed Mexico's signature onto the North American Free Trade Agreement (NAFTA) and its membership as an Organization for Economic Cooperation and Development (OECD) country. As a byproduct of its rapid industrialization, Mexico has experienced an especially rapid increase in the number and scope of environmental exposures experienced by children. Concentrations of ozone, nitrogen dioxide and particulate matter in urban areas now frequently exceed international safety thresholds [1]. Chemical, biological contamination of major sources of drinking water has been reported to increase by 40% over the past 10 years [2-4]. While DDT use has been restricted in Mexico, pesticide metabolites have been documented to persist in at least one survey in Chiapas [5-7].

Mexico's 43 million children, who represent approximately 44% of its total population, experience especially high prevalence and incidence of chronic conditions associated with hazardous environmental exposures. Increases in the incidence of acute lymphocytic leukemia in children have been observed in Mexico between 1982-1991 [8], and this increase has been sustained over the most recent period analyzed, 1996-2002 [9,10]. Among the chemicals linked to childhood cancer are benzene, 1,3-butadiene, and pesticides [11-14]. While asthma prevalence in some areas of Mexico remains below other Latin American countries [15], asthma prevalence varies widely within each region [16]. Differences in these rates may be related to differences in ozone and other outdoor air pollutants which have been linked to the exacerbation and development of asthma [17-23].

The National Academy of Sciences has documented generic vulnerabilities of children to environmental hazards [24]. Many children have additional risk factors that further heighten their vulnerability to chemical factors commonly observed in the environment. More than 3 million Mexican children aged 6 to 14 years work under hazardous conditions, despite the fact that the Constitution establishes 14 years as the minimum age for work [25]. Most of these children live in remote villages, with limited access to health services [26]. The National Program of Agricultural Day Laborers (Programa Nacional de Jornaleros Agrícolas) estimates that Mexico has 500,000 child farm workers are at increased risk for pesticide exposure [6,27,28]. Take-home exposures among children whose parents use lead occupationally remains a major concern [29]. Undernutrition remains highly prevalent among preschool children, with caloric deficiency as common as 30% nationally [30].

Regulation of environmental chemicals has proven successful in the reduction of childhood disease and disability [31]. Reductions in exposure associated with the elimination from lead in gasoline in the United States resulted in IQs among preschool aged children in the 1990s that were 2.2-4.7 points higher than they would have been if those children had a distribution of blood lead levels found among children in the 1970's [32]. Before the US Environmental Protection Agency's (USEPA) phase out of diazinon and chlorpyrifos, these two pesticides were frequently detected in the cord blood of New York City children and associated with decrements in birth weight and length. After these phase outs, the pesticides and their association with predictors of cognitive potential were no longer detected [33]. Restrictions instituted by the city of Atlanta on vehicular travel during the 2000 Olympic Games were associated with significant reductions in ambient ozone and in asthma acute care events [34].

Though the importance of prevention, education and advocacy to reduce environmental health risks has been well described [35], few Organization for Economic Cooperation and Development (OECD) governments have translated scientific understanding of children's unique vulnerability into environmental policy action [36]. Increases in chronic conditions among Mexican children suggest major opportunities for implementation of interventions to reduce environmental risk and childhood disease, but no study has assessed the state of governance instruments with regard to children's environmental health in Mexico.

We therefore decided to systematically review governance instruments concerned with protection of children from environmental exposures in Mexico. We complemented these data with interviews to provide a more complete analysis of the major policy gaps that exist in the protection of children from environmental hazards in Mexico. Our ultimate goal through this process was to provide recommendations to Mexican government officials, scientists and nongovernmental organizations (NGOs) that would ultimately produce reductions in disease and disability of environmental origin in Mexican children.

## Methods

The study protocol was reviewed and approved by the Research Review Board and the Ethics Committee at the National Institute of Public Health (INSP, Mexico) in 2007. We applied the World Health Organization (WHO) definition of environmental health for purposes of this analysis. This definition "addresses all the physical, chemical, and biological factors external to a person, and all the related factors impacting behaviours" and "excludes behaviour not related to environment, as well as behaviour related to the social and cultural environment, and genetics" [37].

We used a two-step approach to our analysis. First, we utilized a systematic review to identify the relevant international and national governance instruments and policy tools to which Mexico adheres relating to children's environmental health. We analyzed PubMed and governmental and NGO reports obtained from the Web sites of the World Health Organization (WHO), United Nations (UN), Commission for Environmental Cooperation (CEC), the Organization for Economic Cooperation and Development (OECD) and the North American Agreement on Environmental Cooperation (NAAEC), the main NAFTA parallel environmental accord, to identify regulations regarding children's environmental health to which Mexico might adhere. Our approach to identifying governance instruments (GIs) from the above Web sites involved searching for the following keywords: children, infants, fetus and pregnant women. We used the same words to search Mexican legal code, as well as websites of the Secretariat of the Environment and Natural Resources (SEMARNAT), the Ministry of Health (MoH), the Federal Commission for the Protection against Sanitary Risk (COFEPRIS), the National Ecology Institute (INE) and the Secretariat of Social Development (SEDESOL).

Having identified the scope of possibly relevant governance instruments, one of the authors (E.C.) screened these GIs for their relevance in protecting children from environmental hazards in Mexico. International and Mexican GI's that remained were more carefully scrutinized by two of the authors (E.C. and L.T.) for the degree to which they may protect children from environmental hazards. In the results section we present descriptive results of those GI's that met these search criteria, and provide the analytic perspectives of the authors.

The second component of our analysis consisted of a series of interviews with high-level public officials, researchers, and representatives of non-governmental organizations (NGOs). Potential interviewees were identified by using policy mapping procedures (i.e., role in government), critical appraisal of literature (using PubMed), and snowballing techniques. Eligible informants were identified following a purposive sampling scheme, the main purpose of which was to obtain diversity of interviewees, rather than a representative sample [38]. A total of seventeen key informants were invited to participate and received an advance script of the interview, along with the objectives and an introductory explanation of the research. Fifteen agreed to participate in the study and provided written informed consent: seven interviews were completed with high-level public officials (two from MoH; two from SEMARNAT; two from INE and one from SEDESOL) involved in regulatory activities, enforcement and social policy, respectively. Six interviewees were recruited from universities and public health institutions, and the last two were members of non-governmental organizations (NGOs). One high-ranking official (COFEPRIS) and one NGO representative never replied to our repeated calls.

Our review of the relevant international and Mexican GI's assisted us in developing content for the structured interviews around emerging issues, major policy actors and connections between them [39,40]. An annotated outline was developed along with interview guidelines focusing on three major themes: policy tools, gaps and potential recommendations. Each individual interviewee was approached separately, and all interviews were conducted in a private environment by two of the authors (MR and EC) who had no direct working relationship with the interviewees. All but two interviews were recorded and transcribed; when interviewees refused to be recorded, careful notes were taken instead. Interviewees chose whether to be identified by name or remain completely confidential. Each interview took less than 45 minutes to be completed.

Responses were sorted according to the major themes described above. Analysis of the interview transcripts progressed towards the identification of overarching themes that captured the phenomenon of governance and policy making feedback, as described by key informants [41]. We coded themes according to major policy actors and connections between them, emerging issues, gaps and feasible recommendations [39,40]. Following a combined technique of inductive and deductive thematic analysis [42,43], we progressed towards the identification of the phenomenon of governance in the national context.

## Results

### Analysis of International Governance Instruments

We reviewed nearly 690 international policy tools that contained any of the required search terms. Most of these tools address chemicals, air quality, second hand tobacco smoke, food safety, and a growing list of environmental risks on a population-wide basis. However, we identified only two of these governance instruments (GI's), both from the United States, that make emphasis on the unique vulnerability of children with regard to environmental hazards [44,45]. No international GI that Mexico upholds reflects the unique vulnerability of children with regard to environmental hazards.

### Analysis of Mexican Governance Instruments

We identified two Mexican governance instruments (GI's) which address children's environmental health, but only in very broad terms. The two GI's were the Mexican Constitution and the General Law on Health (Ley General de Salud). The former establishes that every individual has the right to health protection and also declares the right of children to satisfy their nutritional, health, educational, and recreational needs [46,47]. The General Law on Health (GLH) sets forth the objectives of the right to health protection as well as the objectives of the National Health System; GLH chapter 5 defines maternal and child care and focuses on the protection of children, activities to support families and contribute to maternal and child health, appropriate standards of school hygiene, and health services for schoolchildren. The Law on the Protection of the Rights of Children and Adolescents [48], includes a set of guidelines concerning health promotion for school children.

As part of the North American Free Trade Agreement (NAFTA) and parallel accords, the Mexican legislature passed the Law of Norms and Harmonization ("Ley de Metrologia y Normalizacion"), which follows and monitors compliance of Canada, US and Mexico obligations. This law of harmonization resulted in new *Normas Oficiales Mexicanas *(we translate *Normas *as "norms", though they have more political force than the English word suggests). These norms were formalized in the Law on Ecological Equilibrium and Environmental Protection [49].

Our review of these tools showed that regulatory agencies are increasingly relying on data derived from risk assessment, risk management and epidemiological data to inform and reinforce environmental health policy. The regulatory initiatives to promote standard methods for the evaluation of environmental hazards and establishment of maximum permissible limits for environmental contaminants, however, do not specifically address children's unique vulnerability or particular health needs. Only four *Normas *included any of our search terms (Table 1). Three of these mention children's unique vulnerability to carbon monoxide, ozone and particulate matter, but do not provide further details, assess additional safety factors or modify regulations in light of this vulnerability. The ozone safety threshold (0.110 ppm) is higher than the one in the United States (0.075 ppm) that was recently modified in light of new scientific data about health effects in children [50]. Regulations on pesticides, food additives and drinking water contaminants make no mention whatsoever of children.

**Table 1:** Selected examples of environmental policy tools in Mexico

AIR		
***NORM ***	***Brief description***	***CHILDREN ***

Carbon Monoxide (CO). NOM-021-SSA1-1993	Mexican government requires catalytic converters on cars since 1990, thus dramatically reducing carbon monoxide and hydrocarbon emissions.	Mentions children's vulnerability. No further details are provided
Ozone (O_3_). NOM-020-SSA1-1993	The safety threshold was reduced from 0.281 ppm to 0.110 ppm in 2002	Mentions children's health vulnerability. No details are provided
Particulate matter NOM-025-SSA1-1993	Establishes the threshold of maximum concentration of PM10 and PM 2.5	Mentions children's health vulnerability. No details are provided

**WATER**		

Drinking water quality regulations NOM-127-SSA1-1994	Establishes the threshold of biological and chemical contaminants	No mention to children's specific needs

**FOOD**		

Food and beverages related regulations NOM-086-SSA1-1994	Establish overall nutrimental requirements, additives in food and beverages	No mention to children's specific needs

**PESTICIDES**		

Examples: DDT and Chlordane. NOM-032-SSA2-2002	DDT is restricted (not legally prohibited). MoH has discontinued its use (e.g., against malaria) since 2002, and elimination of the use of chlordane since 1998.	No mention to children's specific needs

**LEAD**		

NOM-199-SSA1-2000	See Table 2	Only environmental policy tool concerned with the health specific needs of children or pregnant and lactating women

The fourth of these *Normas *describes (NOM-199-SSA1-2000) in depth specific measures to be taken to reduce developmental neurotoxicity and other health effects from lead (Table 2). Mexico uses an action level of 10 μg/dL for childhood blood lead [51], which is similar to guidelines promulgated by the US Centers for Disease Control and Prevention, though studies have suggested that effects on cognition emerge at even lower levels in children [52,53]. Guidelines for reassessing blood lead and reducing preventable exposures generally follow current American Academy of Pediatrics guidelines [54]. It is worthy to note that the guidelines for management of lead poisoning mention specifically glazed pottery and other sources which are uniquely associated with neurotoxic exposure in this population [55,56].

**Table 2:** Lead and children's health policy (NOM-199-SSA1-2000) in Mexico

Blood lead levels (BLL)	Children < 3 years	Children 3 - 15 yrs	Pregnant and breast feeding women
< 10 μg/dL	NO ACTION REQUIRED	NO ACTION REQUIRED	NO ACTION REQUIRED
10 - 14 μg/dL	Follow up (BLL). Inform parents (BLL results) Identify sources of exposure Hygiene and nutritional advice	Follow up (BLL). Inform parents (BLL results) Identify sources of exposure Hygiene and nutritional advice	Inform BLL results Identify sources of exposure Follow up (Mother and child) Hygiene and nutritional advice
15 - 24 μg/dL	Repeat venous blood test periodically until BLL < 10 μg/dL Prescribe nutritional supplementation (Iron, Calcium) Assess BLL among family members (<15 years, pregnant and breast feeding women). Identify sources of exposure. Notification to the Health Authority. Protection measures (e.g., glazed pottery removal). Hygiene and nutritional advice	Repeat venous blood test periodically until BLL < 10 μg/dL Prescribe nutritional supplementation (Iron, Calcium) Assess BLL among family members (<15 years, pregnant and breast feeding women). Identify sources of exposure. Notification to the Health Authority. Protection measures (e.g., glazed pottery removal). Hygiene and nutritional advice	Repeat venous blood test until breast feeding period is concluded and BLL< 10 μg/dL Prescribe nutritional supplementation (Iron, Calcium) Assess BLL among family members (<15 years, pregnant and breast feeding women). Identify sources of exposure. Notification to the Health Authority. Protection measures (e.g., glazed pottery removal). Hygiene and nutritional advice
25 - 44 μg/dL	Monthly monitoring (venous blood test) until BLL< 25 μg/dL Case management and nutritional supplementation (Iron, Calcium) Assess BLL among family members. Identify sources of exposure. Notification to the Health Authority. Protection measures (e.g., glazed pottery removal). Hygiene and nutritional advice	Bi-monthly monitoring (venous blood test) until BLL< 25 μg/dL Case management and nutritional supplementation (Iron, Calcium) Assess BLL among family members. Identify sources of exposure. Notification to the Health Authority. Protection measures (e.g., glazed pottery removal). Hygiene and nutritional advice	Monthly monitoring (venous blood test) until BLL< 25 μg/dL or breast feeding period is concluded Case management and nutritional supplementation (Iron, Calcium). Identify sources of exposure and notification to the Health Authority. Assess BLL in cord blood. Assess BLL among family members. Protection measures (e.g., glazed pottery removal). Hygiene and nutritional advice Follow up mother-child
45 - 69 μg/dL	Recommendations similar to above category. In addition, chelating agents, under strict medical supervision, until BLL <45 μg/dL. Referral to social worker, if necessary	Recommendations similar to above category. In addition, chelating agents, under strict medical supervision, until BLL <45 μg/dL. Referral to social worker, if necessary	Recommendations similar to above category. Notification to Health Officer; referral to specialized health care within the next 48 hrs and repeat BLL test. No pharmacological treatment during pregnancy. Careful decision after delivery.
> 70 μg/dL	Emergency case. Recommended procedures are similar to above category (<69 μg/dL) Specialized medical care	Emergency case. Recommended procedures are similar to above category (<69 μg/dL) Specialized medical care	Emergency case. Recommended procedures are similar to above category (<69 μg/dL) Specialized medical care and careful decision regarding treatment.

### Interviews

According to most interviewees, Mexico's governance and implementation of environmental policy concerning children's health are lagging behind. Practically every high level official interviewed pointed out that Mexico is still focusing on improving public health overall, rather than focusing on policies to protect children from environmental hazards. We found repeated concerns regarding State centralization of power that pose major institutional barriers to social participation; these barriers are reflected in the extent in which political power is highly concentrated in the executive branch, and the relatively minor role that political parties and the legislative branch play in the decision making process.

Eleven of the fifteen interviewees were aware of Mexican environmental policy tools addressing children's health needs. The overall consensus among the researchers interviewed was that, with few exceptions (lead was often quoted), maximum permissible thresholds for environmental contaminants do not address the specific health needs of fetuses and pregnant women. Researchers consistently stated that Mexico's environmental policy tools concerned with children's health are lagging behind and that the youngest citizens' health needs have been traditionally relegated to the background in the environmental policy agenda. Advocates argued that Mexico's environmental policy is insufficient to protect children's health, and that the government agencies are often to slow to react to evidence showing how pollution is affecting children or too unwilling to inform the public about environmental risk for children. There was consensus was that legislative weakness has contributed to the absence of efforts to protect its youngest citizens.

Officials from the Ministry of Health (MoH) did highlight recent *Normas *concerning air quality, lead, toys and pesticides (e.g., DDT) as examples of positive progress to protect children, but some did agree that measures to protect children from environmental hazards were lagging behind. One of them noted that structural gaps often make it difficult to build operational bridges, mobilize other sectors, and reinforce the whole process. Another MoH official voiced apprehension that the private sector commonly views children simply as small adults. Most high-ranking officials cited state centralization of power as well as visible cuts and financial constraints when discussing intersectoral coordination and gaps. Two of the interviewees from this group said they did not believe that evidence on particular vulnerabilities of children to environmental exposures should inform health protection policies and protection programs, and when asked for a rationale, one of them simply stated that environmental hazards for adults are also hazardous for children and quoted a short list of examples (second hand tobacco smoke, unhealthy food environments and lack of facilities for physical activity, water pollution and climate change).

When asked about the role of research in informing policy to protect children, high-level government officials quoted political inertia, increasing cuts and multiple legislative gaps as barriers to policy development and implementation. The overall perception regarding opportunities was clearly described by a high ranking informant who summarized the difficulties posed by weak laws, growing environmental challenges and decreasing budgets. When asked about links with researchers and communication barriers, she continued by stating that most Mexican researchers from the environmental and public health related disciplines are motivated by academic prestige attached to data publishing in scientific journals, instead of translating knowledge into lay language and advocacy tools.

An exception to this phenomenon was noted with regard to lead poisoning. In part, efforts to prevent lead poisoning were made more publicly palatable by the catastrophe that which occurred in mid-1999, when more than 1,000 children in the desert town of Torreon, some 500 miles (800 km) north-northwest of the Mexican capital, were found to have dangerously high blood lead levels, which authorities linked to emissions from a local metals smelter. The furor over the contamination prompted an unprecedented government-ordered cleanup by the mining giant Peñoles, a move hailed as a landmark in reversing the impunity industrial polluters long enjoyed in Mexico [57,58]. Further epidemiological studies, conducted during the early 1990s, identified additional sources of exposure: leaded gasoline, as well as use of lead-glazed ceramics for cooking and food storage. In response, public health efforts addressed effective prevention of lead exposures for pregnant women and fetuses [59-62]. Two high-level public officials from the Secretariat of the Environment and Natural Resources (SEMARNAT) recommended that different sectors should work together, updating policy and encouraging translational research and evidence-based health interventions.

Key informants from non-governmental organizations in Mexico noted that Mexico's advocacy movements to protect children from environmental hazards are still in their infancy. Both interviewees echoed statements concerning centralization of power, major political barriers and obstacles to social participation. One interviewee indicated that changes brought about by the MoH during the last administration have created a new bureaucracy and a series of policy gaps, leaving children even more unprotected than ever before. A fundamental reason, he explained, is that policy tools and initiatives may exist, but they are often part of an enormous facade, only to give the impression that something is being done but, when analyzed from the inside, nothing is actually happening. The other informant stated that political will does not exist beyond rhetoric speeches and formal regulations. When we asked her for potential recommendations, she simply replied that it is not easy to implement feasible measures, when most economic and human resources are increasingly diverted to fight organized crime, weapons and illicit drugs, instead of investing in children's health and environmental policy.

Researchers emphasized that evidence concerning environmental pollution and children health is considerably stronger today than one decade ago, and practically everyone pointed out the increasing number of studies from industrialized countries as well as a growing body of research conducted in Mexico, Brazil, Chile and other Latin American countries. Half of this group of interviewees recognized that they had never participated in policy oriented discussions or advocacy related activities. When asked why, they simply answered that it was not their job to do so or that policy makers are seldom (or not always) willing to consider evidence as input for implementation of regulation and health protection measures. Two senior researchers said they had witnessed slow progress because of weak institutional interest in translational research and the lack of explicit and well defined communication channels between scientists and policy makers. One of them referred to a divorce between legislators and children's environmental health concerned researchers and the other concluded that both researchers and legislators were responsible for poor translation.

## Discussion

The primary finding of this study is that the environmental legal framework in Mexico is not yet linked with the growing body of evidence regarding both the biological and social vulnerabilities of children to environmental hazards. Similar challenges in modifying legislation to protect children have been reported in US and Canada [35,36]. To our knowledge, this is the first assessment of the environmental policy and children's health conducted in Mexico. While our considerations focus on Mexico, the methodology applied in this case study may be appropriate also in regard to other countries to examine adequacy of policy instruments to protect children from environmental hazards.

The study was conducted immediately at the end of the Vicente Fox administration (2000-2006), which came to power after defeating the ruling party (the Institutional Revolutionary Party, or PRI, had remained in power for the preceding 70 years), while creating great expectations for governmental reform. Our investigation showed that even today, there is no coherent policy, and new challenges exist in the current era of financial constraints and government downsizing. We identified a series of communication barriers between researchers and policy decision makers, built upon institutional culture and mutual mistrust. On the one hand, most research groups restrict their role to knowledge production, while focusing only on publication of results, rather than translational advocacy; the majority of public officials involved in policy making process, on the other hand, tend to disregard scientific evidence regarding both the high prevalence and increasing incidence of pediatric diseases associated with environmental exposures. Not surprisingly, for example, no laws addressing housing conditions were found, despite the fact that young children spend most of their lives indoors.

Furthermore, advocacy has had a weak impact, if any, on health protection measures in part because children's environmental health is not on the radar screen of many Mexico's NGOs. Mexico is not, however, totally devoid of non-governmental groups or community groups that are concerned about children's environmental health issues. The Commission for Environmental Cooperation has awarded a number of grants through its North American Fund for Environmental Cooperation to organizations that demonstrate sensitivity to children's health and the environment, such as the Instituto de Culturas Nativas de Baja California and the Instituto de la Naturaleza y la Sociedad de Oaxaca [63].

Lack of information about environmental risk factors represents a major barrier for many families and communities. Of paramount importance is informing communities of concern about approaches to minimize hazards from a growing list of environmental contaminants. In addition to increasing efforts by nongovernmental organizations to communicate about children's environmental health issues, the press is giving increasing attention to risks posed by chemical exposures and children, such as those experienced by San Quintin farmworker families in Baja California Norte [64]. Major conferences in Latin America have also served to highlight the need to focus on children's health and the environment. This includes a meeting of Health and Environment Ministers of the Americas, which met in Mar del Plata, Argentina on 16-17 June 2005. We do not dispute that NGOs and governmental agencies are not working to improve awareness of these issues, but do suggest that serious gaps in knowledge persist and merit further intervention.

As with any qualitative research study, there are important caveats to be made. Our systematic review endeavored to be as complete as possible, though we may have omitted sources of information that may underreport to some degree the scope of governance instruments and policies intended to protect children from environmental hazards. Indeed, our interview process is based upon a small number of responses and respondent bias, as well as concern about confidentiality of responses, may have limited the depth with which certain respondents commented. Nonetheless, together these two data sources provide insights never before obtained about a developing country amidst significant industrialization and increasing scientific understanding of the impact of environmental hazards on children's lives. Despite these limitations, our results strongly suggest that significant gaps remain in the system developed to protect children from environmental hazards in a country that is likely to experience increases in scope of industrialization and potentially hazardous environmental exposures.

The challenge Mexico faces in the future is to design policies that specifically protect children against environmental exposures. A systematic review of US state policies has identified a series of model environmental regulations that can be used to prevent neurodevelopmental disabilities and asthma in children [65]. These include: reductions in mercury emissions from coal-fired power plants, bans on smoking in public places, incentives for implementation of integrated pest management (IPM), limits on arsenic contamination in drinking water, limits on diesel vehicle idling, and requirements to reduce volatile organic compound use in household products. While these model policies may require some modification for use in the Mexican national context, they do serve as bases for the implementation of proactive measures to limit harmful exposures.

While identification of model policies is a useful first step, the social will must also exist to implement them. In the United States and Europe, progress towards protecting children from environmental risk factors has emerged out of a joint effort of academic researchers, government officials and advocates in translating knowledge from theory to population impact. While a WHO Collaborating Center in Children's Environmental Health does exist at the University of San Luis Potosi, resources need to be established across other parts Mexico to ensure effective dissemination of knowledge about environmental risk factors across this large and geographically dispersed population. One model for the effective dissemination of knowledge about environmental risk factors is the establishment of regionalized Pediatric Environmental Health Specialty Units (PEHSU) where researchers and clinicians work together to identify, study and remediate local population concerns.

Indeed, one PEHSU was established at the National Institute of Public Health and Children's Hospital of Morelos with support from the EPA (2001 to 2008)  in concert with the CEC trilateral program on children and environment, but has subsequently had its stream of funding terminated. Before its termination, this PEHSU built an impressive track record of communication locally and nationally about a host of hazards, ranging from water contamination to unhealthy food. Government officials should recognize this track record of success and must devote additional resources to children's environmental health in Mexico. Further fiscal support for clinical and research facilities is needed to document burden of environmentally-mediated diseases in Mexican children and identify preventable risk factors for intervention by policy makers, communities and clinicians. While competing public health priorities exist, expanding efforts in children's health beyond the prevention of infectious illnesses would stem the tide of an increasing epidemic of chronic disease in Mexican children [5-7,20,66,67].

Even if resources are established to study, prevent and treat diseases of environmental origin, improving communication between the research and advocacy communities was identified as a major concern across many of the interviews. Whether or not PEHSUs are reestablished in Mexico, researchers should establish more collaborative modes of communication with the government officials and the advocacy community to ensure effective and appropriate translation of research findings. This can easily be done in a manner that does not compromise scientific integrity.

Strong concerns were raised about threats of economic development as a barrier to proactive protection of children from environmental hazards. Perceptions can cloud reality, especially when economic progress is primal in the minds and hearts of government officials. Examples from the systematic review of US states [65] include many initiatives that do not come at a cost to economic progress. These include widespread use of integrated pest management, which in Ecuador, reduced pesticide applications and lowered the overall amount of pesticides needed. The IPM fields yielded as many or more potatoes but production costs decreased from USD$104/ton potatoes produced to USD$80/ton, while neurological effects among farmers and their families decreased [68]. Examples like these must be communicated along with the economic benefits associated with reduced disease and disability among children when proactive regulation is instituted to limit toxic environmental exposures. Removal of lead in gasoline is likely to have resulted in similar economic benefits in Mexico to that achieved in the United States, where annual economic productivity of each birth cohort increased by $110-319 billion [32].

We were surprised that international treaties did not support ongoing efforts to improve children's environmental health in Mexico. Indeed, as NAFTA continues to open trade across North America, children in all three countries will be exposed to contaminants in products made in any one of them. While gaps do remain in US policy with regard to children and protecting them from environmental hazards [65], our review identified opportunities for US leadership that would be of mutual benefit to Mexican and US children. It is important to recognize the ongoing effort of the Commission for Environmental Cooperation (CEC), a trinational organization that has partnered with each North American government to issue a first report on children's health and the environment [69]. We would encourage CEC to include policy outcomes in future reports, and ensure effective communication of successful initiatives and lessons learned in policy initiatives to protect children.

As Mexico moves through the twenty-first century, concerns regarding environmental exposures loom globally. In 2020, the developing world will account for 33% of world chemical demand and 31% of production, compared with 23% and 21% respectively in 1995 [70]. It is equally imperative to develop and implement policies across industrializing countries and less developed nations around the globe to protect the health of our children now and in the future. As Mexico and other countries emerge as developed economies, industrialization is likely to result in broader and greater exposures to industrial chemicals. If regulatory efforts are not taken to limit environmental exposures that are known or suspected to be hazardous, industrialization could result in increases in environmental exposure to toxic chemicals which have been linked to epidemics of chronic conditions in the United States and other developed countries [31,71]. Children are arguably a country's greatest natural resource, and policies to protect children from environmental hazards can have great long-term benefits, given their greater future years of productive life. Mexico, like other countries, will continue to wrestle with decisions to distribute resources towards improvement of public health of its entire population rather than focus those resources on children and other vulnerable populations. However, many policy options to improve protections of children would not require governmental resources, and yield economic benefits many times over for decades to come.

## Conclusions

Policy tools to protect children from environmental hazards in Mexico are lacking, with the possible exception of lead. Opportunities exist to prevent toxic environmental exposures and chronic childhood conditions in Mexico if proactive policy measures are developed and implemented.

## Abbreviations

CEC: Commission for Environmental Cooperation of North America; COFEPRIS: Federal Commission for the Protection against Sanitary Risk; GI's: Governance Instruments; IPM: Integrated Pest Management; INE: National Institute of Ecology; INEGI: National Institute of Statistics and Geography; MoH: Ministry of Health; NAAEC: North American Agreement on Environmental Cooperation; NAFTA: North American Free Trade Agreement; NGOs: Non Governmental Organizations; OECD: Organization for Economic Co-operation and Development; SEDESOL: Secretariat of Social Development; SEMARNAT: Secretariat of the Environment and Natural Resources; USEPA: United States Environmental Protection Agency; WHO: World Health Organization.

## Competing interests

The authors declare that they have no competing interests.

## Authors' contributions

EC conceived the study and obtained primary funding. EC and MR collected the primary data. Analysis was performed by EC and LT. LT, EC, MR and PJL reviewed and refined the manuscript. All authors read and approved the final manuscript.
